# Effects of state immigrant insurance coverage policies on access to adequate prenatal care among immigrant pregnant women in the United States

**DOI:** 10.1016/j.jmh.2025.100350

**Published:** 2025-07-27

**Authors:** Gunah Kim, S. Wilton Choi, Younhee Kim

**Affiliations:** a Department of Health Policy and Management, College of Health Science, Korea University, Seoul, Republic of Korea, 145 Anam-ro, Seongbuk-gu, Seoul, Republic of Korea; b School of Public Health, College of Health and Human Services, San Diego State University, 5500 Campanile Drive, San Diego, CA 92182, USA; c School of Public Affairs, Pennsylvania State University Harrisburg, 777 W. Harrisburg Pike, Middletown, PA, USA

**Keywords:** State immigrant insurance coverage policies, Adequacy of prenatal care: prenatal care initiation, Immigrant health, Maternal and infant health, Health equity

## Abstract

**Background:**

The 1996 federal welfare and immigration reform restricted immigrant eligibility for public health insurance such as Medicaid and CHIP. As of January 2023, 34 states have adopted policies to expand insurance coverage for immigrant pregnant individuals through Medicaid/CHIP.

**Objective:**

To estimate the effects of state immigrant insurance policies on prenatal care utilization and timing among pregnant immigrants.

**Methods:**

A difference-in-differences approach was used to compare states that expanded immigrant insurance coverage to those that did not. The main data source is the restricted natality data from the National Center for Health Statistics, including all singleton births to immigrant mothers aged 15–44 across all 50 states and D.C. from 2015 to 2019.

**Results:**

In states adopting the State-only funds option, publicly insured immigrants had higher odds of receiving intermediate (OR: 1.429; 95 % CI: 1.210–1.687), adequate (OR: 1.723; 95 % CI: 1.526–1.946), and adequate plus (OR: 1.373; 95 % CI: 1.256–1.500) prenatal care, and lower odds of inadequate care (OR: 0.480; 95 % CI: 0.406–0.568) compared to uninsured immigrants. Additionally, this policy was associated with an 87.1 percentage point increase in first-trimester care initiation (95 % CI: 1.622–2.159), and significant decreases in delayed care (−43.8 pp; 95 % CI: 0.430–0.736) and no care until delivery (−67.3 pp; 95 % CI: 0.204–0.522) for publicly insured immigrant populations compared to uninsured immigrants.

**Conclusions:**

Expanding immigrant insurance coverage was associated with earlier and more adequate prenatal care. However, only State-only funds showed consistent improvements in the adequacy of prenatal care utilization.

## 1 Introduction

The U. S. maternal mortality rate has shown a concerning upward trend, reaching record highs in 2021 (Hoyert, 2023), with disparities by nativity status persisting (Government Accountability Office, 2004; Brown et al., 2007; Cervantes et al., 1999; Collins and Shay, 1994; Collins and David, 2009; Fuentes-Afflick and Lurie, 1997). Research claimed that these poor maternal health outcomes attributed to in adequate prenatal care utilization (Louis and Platt, 2011; Kotelchuck, 1994), which is essential for identifying health risks to both mother and baby and preventing other potential complications (American College of Obstetricians and Gynecologists, 2014a; Child Trends Databank, 2015; Partridge et al., 2012). Thus, timely and adequate prenatal care can reduce the risk of adverse pregnancy-related health outcomes.

Access to health insurance is critical for healthcare accessibility, yet it remains a biggest barrier for vulnerable populations, leading to health disparities, especially among low-income families and minority groups (Call et al., 2014; Hadley, 2003; Franks et al., 1993; Zhu et al., 2010; DeNavasWalt, 2011; Majerol, 2015; Nelson, 2002). Uninsured individuals are more likely to delay necessary care due to high out-of-pocket costs, which reduces their likelihood of receiving necessary preventive healthcare services, such as prenatal care (Board on Health Care Services, Committee on Health Insurance Status, & Its Consequences, 2009). An extensive literature suggested that expanded health insurance coverage by policy adoptions improves access to prenatal care among minority groups and immigrant families (Janevic et al., 2022; Dubay et al., 2001; Eliason and Daw, 2022; Drewry et al., 2015; Palmer, 2020; Gavin et al., 2007; Miller et al., 2022; Bustamante et al., 2019; Alcalá et al., 2017; Lyu and Wehby, 2021).

The Personal Responsibility and Work Opportunity Act (PRWORA) of 1996 limited eligibility to federal safety-net programs, like Supplemental Nutrition Assistance Program (SNAP), Medicaid, and State Children’s Health Insurance Program (CHIP), for the most of legal immigrants, affecting their ability to obtain health services (Parmet et al., 2017; Singer, 2004). In response, several states have implemented policies to counteract the reverse effects of PRWORA by restoring eligibility for these programs, particularly for immigrant children and pregnant women. Key policies include the Legal Immigrant Children’s Health Improvement Act (ICHIA) of 2007, incorporated into the Children’s Health Insurance Program Reauthorization Act (CHIPRA) of 2009. Under the section 214 of CHIPRA, states can opt to enroll lawfully present immigrant children and pregnant women in Medicaid and CHIP regardless of their five-year waiting period (Ku and Blaney, 2000).

Another state policy, From-Conception-to-the-End-of-Pregnancy (FCEP), previously known as the unborn child option, uses CHIP funds to extend coverage to pregnant women who are not income-eligible for Medicaid from conception to birth (Jarlenski et al., 2014), providing a simplified enrollment process without residency proof (Sperow, 2004). Additionally, several states allocate state-only funds to cover income-eligible pregnant women who are otherwise ineligible due to immigration status after welfare reform in 1996 (Brooks et al., 2021; Wherry et al., 2017). For instance, the DC Healthcare Alliance Program, which is a locally-funded program, provides medical assistance to immigrants residing in the District of Columbia (Department of Health Care Finance, 2022).

While numerous studies have investigated the effects of policies providing public health insurance coverage to populations with restricted access (Saloner, 2013; Huang et al., 2006; Daw and Sommers, 2018, 2019; Wherry, 2018), there is limited research specifically addressing the impact of three policy options critical for immigrant pregnant women on expanding health insurance coverage for pregnant immigrant women. This study addresses this gap by estimating the effects of state policy adoptions on the initiation and adequacy of prenatal care among immigrant families, with using the recent population based birth records that covers all newborns from immigrant families in the United States. Through this comparative analysis, the study aims to clarify the impact of Medicaid, CHIP, and state-funded policies on prenatal care accessibility, providing insights into how state-level health insurance expansions can improve health outcomes for immigrant populations.

## 2 Data and methods

### 2.1 Data and population

This study used the restricted natality dataset from the National Center for Health Statistics (NCHS) for the years 2015–2019, which includes records of all registered births based on birth certificate data from 50 states, the District of Columbia, and U.S. territories (National Center for Health Statistics, 2019). This dataset provides extensive details on each birth, covering maternal and infant characteristics. The sample consisted of all singleton births to immigrant mothers aged 15–44, born in hospitals across in the 50 states and the District of Columbia between January 2015 to December 2019. Immigrant mothers are defined as foreign-born US residents (Duleep and Dowhan, 2002). The final analytic sample included 2333,526 mothers and infants.

### 2.2 Primary outcome measures

The primary outcomes of interest were the adequacy of prenatal care utilization and the timing of prenatal care initiation. We assessed a binary outcome for prenatal care adequacy using the Adequacy of Prenatal Care Utilization Index (APNCU) (Kotelchuck, 1994), which evaluates prenatal care based on both the month of initiation and the adequacy of visit frequency relative to gestational length. This study used the APNCU index to classify the adequacy of prenatal care into four categories based on the timing of initiation and the proportion of recommended visits completed, as defined by the American College of Obstetricians and Gynecologists (American College of Obstetricians and Gynecologists, 2014b). Inadequate care refers to prenatal care that begins after the fourth month of pregnancy or involves fewer than 50 % of recommended visits. Intermediate care indicates the prenatal care initiation by the fourth month and completion of 50 % to 79 % of recommended visits. Adequate care denotes the prenatal care initiation by the fourth month and completion of 80 % to 109 % of the recommended visits. Adequate Plus care represents the prenatal care initiation by the fourth month and receipt of 110 % or more of the recommended visits. The proportion of visits is calculated from the point prenatal care began until delivery. We also examined four binary outcomes concerning the timing of prenatal care initiation, defined by the gestational trimester when care commenced: (1) first trimester (1st to 3rd month), (2) second trimester (4th to 6th month), (3) third trimester (7th month onward), and (4) no prenatal care received until delivery.

### 2.3 Main exposure and risk factors

The main exposures in this study are changes in state adoption of each of the three policy options. According to annual reports by the Kaiser Family Foundation’s annual, which track state-level policy data, a state was considered to have adopted a policy option if it was effect as of January 1 of that year. Besides changes in state policy adoptions, other factors affecting prenatal care utilization included maternal characteristics (age, educational attainment, marital status, race/ethnicity, and insurance type), infant characteristics (birth order and plurality), regional characteristics (county median income and county unemployment rate), and state/year fixed effects.

### 2.4 Data analysis

This study employs a difference-in-differences (DID) approach with fixed effect logistic regression to estimate the effects of insurance coverage policy options on prenatal care utilization among immigrant pregnant women (Angrist and Krueger, 1999). This approach compares changes in prenatal care outcomes among publicly insured immigrant women to those among uninsured immigrant women before and after the adoption, across states implemented the policy and those that did not. This selections assume that publicly insured immigrants are more likely to initiate early prenatal care following policy adoptions than uninsured immigrants. The logistic regression model is estimated as follows:

(1) $$\begin{array}{c} Y_{ijt}=\beta_{0}+\beta_{1}ICHIA_{jt}+\beta_{2}FCEP_{jt}+\beta_{3}StateOnly_{jt}+\beta_{4}Public_{ijt}+\beta_{5}\left(ICHIA_{jt}\times Public_{ijt}\right)+\beta_{6}\left(FCEP_{jt}\times Public_{ijt}\right)\\ +\beta_{7}\left(StateOnly_{jt}\times Public_{ijt}\right)+X_{ijt}+\gamma_{j}+\delta_{t}+u_{ijt} \end{array}$$

As shown in the Eq. (1), $Y_{ijt}$ denotes the outcome, prenatal care utilization, for individual i, in a state j, and in year t. Each policy indicator – ICHIA, FCEP, and State-only – is set to value of one if a state expanded health insurance coverage under the corresponding policy option. $X_{ijt}$ is a vector that includes risk factors outlined above. State (γ_j_) and year (δ_t_) fixed effects were also included to control for unobserved time-invariant state characteristics and time-specific factors.

## 3 Results

### 3.1 Descriptive statistics

Table 1 presents demographic characteristics stratified by the type of supportive policies in the state of residence. Significant differences were observed in outcome measures, as well as in individual and county characteristics of the sample, based on the type of supportive policy. The study included a total of 2333,526 mothers and infants: 1280,847 resided in states with the ICHIA option, 1227,879 in states with the FCEP option, and 341,101 in states with State-only funding options. Those who were residing in a state with any type of supportive policy options were more likely to initiate prenatal care in the first trimester and less likely to have no receipt of prenatal care, compared to those who were living in a state without any of these three options. Those who were living in a state with any type of supportive policy options were less likely to receive inadequate prenatal care and more likely to receive both adequate and adequate plus care, compared to those who were living in a state without any of these three options. Mothers residing in states with any of the three policy options were more likely to have public health insurance and less likely to self-fund delivery costs compared to those in states without such options. Additionally, mothers in states with the ICHIA option and State-only funding were less likely to be of Hispanic, while those in states with the FCEP option were more likely to be Hispanic, compared to their counterparts in states without these respective options. Mothers in states offering both the ICHIA and FCEP options were less likely to be married than those living in a state without those options. Furthermore, median household income and county unemployment rates were higher among those residing in states with the ICHIA option and State-only funds option than among those in states without these policy options.

**Table 1:** Demographic characteristics by the type of supportive policies among immigrant pregnant women (n=2333,526).

	ICHIA Option			FCEP Option			State-Only Funds		
	No (1046,224)	Yes (1280,847)	P value	No (1105,647)	Yes (1227,879)	P value	No (1917,793)	Yes (341,101)	P value
*Initiation of Prenatal Care*									
First trimester	58.88	69.76	< 0.01	61.67	67.75	< 0.01	63.92	70.39	< 0.01
Second trimester	26.87	20.80	< 0.01	25.47	21.79	< 0.01	24.19	19.70	< 0.01
Last trimester	10.10	8.07	< 0.01	9.68	8.35	< 0.01	8.93	9.23	< 0.01
No prenatal care	4.15	1.37	< 0.01	3.18	2.11	< 0.01	2.96	0.68	< 0.01
									
*Adequacy of Prenatal Care*									
Inadequate	29.39	21.02	< 0.01	27.36	22.47	< 0.01	25.49	20.67	< 0.01
Intermediate	11.88	11.56	< 0.01	12.04	11.41	< 0.01	11.62	12.21	< 0.01
Adequate	31.24	37.01	< 0.01	32.44	36.20	< 0.01	33.95	37.17	< 0.01
Adequate Plus	27.48	30.41	< 0.01	28.17	29.93	< 0.01	28.95	29.95	< 0.01
									
*Individual Characteristics*									
Age	29.30	29.62	< 0.01	29.42	29.53	< 0.01	29.40	29.69	< 0.01
Education									
Less than high school	36.94	36.56	< 0.01	35.38	37.96	< 0.01	37.68	31.43	< 0.01
High school grad	32.64	28.85	< 0.01	30.95	30.23	< 0.01	30.85	29.03	< 0.01
Some college	16.98	18.78	< 0.01	18.77	17.22	< 0.01	17.22	22.11	< 0.01
Bachelor's degree	10.02	11.58	< 0.01	11.03	10.73	< 0.01	10.53	12.77	< 0.01
More than college	3.43	4.23	< 0.01	3.88	3.86	0.44	3.72	4.67	< 0.01
Marital status									
Married	55.04	43.59	< 0.01	53.32	44.63	< 0.01	47.65	54.96	< 0.01
Unmarried	44.96	56.41	< 0.01	46.68	55.37	< 0.01	52.35	45.04	< 0.01
Race/Ethnicity									
Hispanic	68.60	59.64	0.10	59.65	67.36	< 0.01	66.60	47.23	< 0.01
Non-Hispanic White	10.19	10.12	< 0.01	10.84	9.53	< 0.01	9.34	14.76	< 0.01
Non-Hispanic Black	11.48	10.98	< 0.01	15.45	7.36	< 0.01	10.23	16.77	< 0.01
Non-Hispanic Asian	8.26	17.75	< 0.01	12.50	14.32	< 0.01	12.25	20.29	< 0.01
Non-Hispanic Other	1.46	1.51	< 0.01	1.56	1.42	< 0.01	1.59	0.94	< 0.01
Insurance type									
Public	78.69	88.26	< 0.01	81.28	86.34	< 0.01	81.44	98.16	< 0.01
Self-pay	21.31	11.74	< 0.01	18.72	13.66	< 0.01	18.56	1.84	< 0.01
Birth order									
First-born	29.00	29.76	< 0.01	30.73	28.24	< 0.01	28.48	34.73	< 0.01
Second-born or more	71.00	70.24	< 0.01	69.27	71.76	< 0.01	71.52	65.27	< 0.01
									
*County Characteristics*									
Household Income	56,710	66,446	< 0.01	61,799	61,787	< 0.01	60,931	65,087	< 0.01
Unemployment	6.90	7.01	< 0.01	6.75	7.11	< 0.01	7.04	6.65	< 0.01

### 3.2 Difference-in-differences results

Table 2 presents the impact of expanding Medicaid and CHIP eligibility on adequacy of prenatal care between publicly insured and uninsured immigrants. The study found that adopting state-only funds policy was significantly associated with higher odds of receiving adequate or adequate plus care among publicly insured immigrants than their uninsured counterparts. Compared to those who are uninsured, mothers with public insurance had increased odds of intermediate care (OR: 1.176; 95 % CI: 1.011,1.367) and decreased odds of adequate care (OR: 0.915; 95 % CI: 0.845,0.991) in states with the ICHIA option. Compared to those who are uninsured, publicly insured immigrants had elevated odds of intermediate (OR: 1.429; 95 % CI: 1.210,1.687), adequate (OR: 1.723; 95 % CI: 1.526,1.946), and adequate plus (OR: 1.373; 95 % CI: 1.256,1.500) care while the odds of inadequate (OR: 0.480; 95 % CI: 0.406,0.568) care were decreased in states with the State-only funds option. However, the estimates suggest that adopting FCEP option was not associated with adequate prenatal care among publicly insured immigrants mothers. The sign of the association was suggestive of positive effects in both intermediate and adequate plus care, but the point estimates were not statistically significant.

**Table 2:** The Effect of Providing Health Insurance Coverage on Adequacy of Prenatal Care Among Immigrant Pregnant Women, 2015–2019 abd.

	Inadequate	Intermediate	Adequate	Adequate Plus
	Care	Care	Care	Care
Publicly insured	0.583⁎⁎⁎	0.772⁎⁎⁎	1.319⁎⁎⁎	1.670⁎⁎⁎
	[0.528,0.643]	[0.670,0.891]	[1.241,1.402]	[1.564,1.784]
ICHIA option	0.985	0.996	1.138*	1.041*
	[0.836,1.160]	[0.873,1.138]	[0.979,1.322]	[0.995,1.089]
FCEP option	1.026	0.670⁎⁎⁎	0.936	1.291⁎⁎⁎
	[0.845,1.246]	[0.582,0.770]	[0.857,1.023]	[1.194,1.396]
State-only funds	2.015⁎⁎⁎	0.752⁎⁎⁎	0.574⁎⁎⁎	0.735⁎⁎⁎
	[1.694,2.396]	[0.642,0.881]	[0.505,0.654]	[0.661,0.818]
Publicly insured X ICHIA option	0.881	1.176⁎⁎	0.915⁎⁎	0.961
	[0.747,1.039]	[1.011,1.367]	[0.845,0.991]	[0.914,1.010]
Publicly insured X FCEP option	0.976	1.028	0.947	1.024
	[0.802,1.187]	[0.879,1.202]	[0.883,1.016]	[0.956,1.097]
Publicly Insured X State-only funds	0.480⁎⁎⁎	1.429⁎⁎⁎	1.723⁎⁎⁎	1.373⁎⁎⁎
	[0.406,0.568]	[1.210,1.687]	[1.526,1.946]	[1.256,1.500]
Mother's age	0.970⁎⁎⁎	0.985⁎⁎⁎	0.999	1.035⁎⁎⁎
	[0.968,0.973]	[0.981,0.989]	[0.998,1.001]	[1.032,1.038]
Highschool	0.734⁎⁎⁎	0.993	1.188⁎⁎⁎	1.092⁎⁎
	[0.673,0.800]	[0.962,1.026]	[1.150,1.227]	[1.008,1.184]
Some College	0.689⁎⁎⁎	0.988	1.214⁎⁎⁎	1.128⁎⁎
	[0.612,0.776]	[0.925,1.056]	[1.152,1.279]	[1.019,1.249]
Bachelor	0.930	0.897⁎⁎	1.144⁎⁎⁎	0.984
	[0.789,1.097]	[0.814,0.987]	[1.086,1.205]	[0.900,1.076]
Master or more	1.037	0.842⁎⁎⁎	1.104⁎⁎⁎	0.957
	[0.858,1.253]	[0.753,0.943]	[1.043,1.168]	[0.839,1.091]
Hispanic	0.903*	0.918⁎⁎⁎	0.929⁎⁎	1.234⁎⁎⁎
	[0.813,1.003]	[0.863,0.977]	[0.864,0.999]	[1.162,1.310]
Non-Hispanic Black	1.690⁎⁎⁎	0.907	0.702⁎⁎⁎	0.910⁎⁎
	[1.429,1.999]	[0.799,1.029]	[0.636,0.774]	[0.836,0.990]
Non-Hispanic Asian	0.930	0.919⁎⁎⁎	1.016	1.103
	[0.839,1.031]	[0.887,0.953]	[0.923,1.120]	[0.969,1.256]
AIAN or NHOPI c	2.145⁎⁎⁎	0.862⁎⁎⁎	0.594⁎⁎⁎	0.791⁎⁎⁎
	[1.801,2.555]	[0.778,0.954]	[0.522,0.676]	[0.690,0.906]
First-born	1.019	0.913*	0.964⁎⁎⁎	1.067⁎⁎⁎
	[0.973,1.067]	[0.830,1.005]	[0.938,0.990]	[1.018,1.119]

a The treatment group is publicly insured immigrants, and the comparison group is uninsured immigrants.

b Odds ratios are reported for all variables. Confidence intervals are in brackets.

c AIAN refers to American Indian or Alaska Native; NHOPI refers to Native Hawaiians and Other Pacific Islanders.

d Year and State Fixed Effects were included but not reported.

⁎ *P* < 0.1 percent.

⁎⁎ *P* < 0.05 percent.

⁎⁎⁎ *P* < 0.01 percent.

It is important to note that the coefficient for the State-only funds variable requires careful interpretation under the difference-in-differences approach. Although it shows a negative association with prenatal care utilization, this parameter reflects the baseline association among uninsured immigrant women in states implementing the policy, rather than the policy's effect on the intended beneficiaries—publicly insured immigrants. Because the model includes interaction terms between policy implementation and insurance status, the coefficient of State-only funds captures the counterfactual trend for the control group. The true treatment effect is identified through the interaction term (Publicly insured × State-only in Table 2, Table 3). Therefore, interpreting the main effect in isolation, without accounting for the interaction structure, may lead to misleading conclusions about policy effectiveness.

**Table 3:** The effect of providing health insurance coverage on prenatal care initiation among immigrant pregnant women, 2015–2019 a^,^^b^^,^^d^^.^

	First Trimester	Second Trimester	Last Trimester	No Prenatal Care
	Initiation	Initiation	Initiation	Until delivery
Publicly insured	1.528⁎⁎⁎	0.953	0.548⁎⁎⁎	0.492⁎⁎⁎
	[1.419,1.646]	[0.890,1.020]	[0.457,0.658]	[0.379,0.640]
ICHIA option	1.039	0.944	1.142	0.855
	[0.920,1.174]	[0.835,1.067]	[0.876,1.489]	[0.506,1.446]
FCEP option	0.881	1.107*	0.790	1.552⁎⁎⁎
	[1.155,1.262]	[0.772,0.843]	[0.888,1.030]	[0.958,1.344]
State-only funds	0.566⁎⁎⁎	0.839*	1.712⁎⁎⁎	2.306⁎⁎⁎
	[0.484,0.662]	[0.701,1.004]	[1.312,2.234]	[1.559,3.413]
Publicly insured X ICHIA option	1.086	1.057	0.718⁎⁎	0.724⁎⁎
	[0.960,1.228]	[0.949,1.178]	[0.539,0.956]	[0.552,0.949]
Publicly insured X FCEP option	0.938	1.344⁎⁎⁎	0.916	0.643⁎⁎⁎
	[0.832,0.931]	[0.983,1.130]	[0.980,1.177]	[0.800,1.089]
Publicly insured X State-only funds	1.871⁎⁎⁎	1.179*	0.562⁎⁎⁎	0.327⁎⁎⁎
	[1.622,2.159]	[0.997,1.394]	[0.430,0.736]	[0.204,0.522]
Mother's age	1.024⁎⁎⁎	0.986⁎⁎⁎	0.976⁎⁎⁎	0.973⁎⁎⁎
	[1.022,1.026]	[0.983,0.989]	[0.972,0.979]	[0.963,0.984]
Highschool	1.359⁎⁎⁎	0.802⁎⁎⁎	0.820⁎⁎⁎	0.659⁎⁎⁎
	[1.246,1.483]	[0.750,0.858]	[0.760,0.885]	[0.572,0.758]
Some College	1.477⁎⁎⁎	0.732⁎⁎⁎	0.847⁎⁎⁎	0.568⁎⁎⁎
	[1.311,1.665]	[0.669,0.801]	[0.765,0.937]	[0.461,0.699]
Bachelor	1.245⁎⁎⁎	0.641⁎⁎⁎	1.572⁎⁎⁎	0.486⁎⁎⁎
	[1.062,1.459]	[0.575,0.714]	[1.349,1.833]	[0.368,0.642]
Master or more	1.198*	0.550⁎⁎⁎	1.979⁎⁎⁎	0.389⁎⁎⁎
	[0.999,1.436]	[0.484,0.624]	[1.612,2.431]	[0.286,0.530]
Hispanic	1.059	1.056	0.810⁎⁎⁎	0.813⁎⁎
	[0.951,1.180]	[0.971,1.150]	[0.709,0.925]	[0.690,0.957]
Non-Hispanic Black	0.583⁎⁎⁎	1.396⁎⁎⁎	1.761⁎⁎⁎	1.181⁎⁎⁎
	[0.498,0.682]	[1.276,1.527]	[1.370,2.262]	[1.069,1.304]
Non-Hispanic Asian	0.988	1.187⁎⁎⁎	0.792⁎⁎⁎	0.731⁎⁎
	[0.895,1.090]	[1.077,1.309]	[0.693,0.904]	[0.537,0.996]
AIAN or NHOPI c	0.501⁎⁎⁎	1.448⁎⁎⁎	1.781⁎⁎⁎	2.026⁎⁎⁎
	[0.423,0.595]	[1.302,1.611]	[1.486,2.133]	[1.657,2.479]
First-born	1.005	0.919⁎⁎⁎	1.155⁎⁎⁎	1.041
	[0.955,1.057]	[0.872,0.969]	[1.103,1.210]	[0.848,1.277]

a The treatment group is publicly insured immigrants, and the comparison group is uninsured immigrants.

b Odds ratios are reported for all variables. Confidence intervals are in brackets.

c AIAN refers to American Indian or Alaska Native; NHOPI refers to Native Hawaiians and Other Pacific Islanders.

d Year and State Fixed Effects were included but not reported.

⁎ *P* < 0.1 percent.

⁎⁎ *P* < 0.05 percent.

⁎⁎⁎ *P* < 0.01 percent.

Maternal characteristics such as educational attainment and first-born status were significantly associated with higher odds of receiving adequate prenatal care. In addition, mother’s race/ethnicity was significantly associated with receipt of adequate prenatal care. Being Hispanic was significantly associated with lower rates of receiving inadequate care and higher rates of receiving adequate plus care. Otherwise, being non-Hispanic Black and being non-Hispanic American Indian or Alaska Native or Native Hawaiians and Other Pacific Islanders were significantly associated with higher rates of receiving inadequate prenatal care and lower rates of adequate or adequate plus care.

Table 3 presents the effect of Medicaid and CHIP eligibility on prenatal care initiation between publicly insured and uninsured immigrants. The findings indicate that adopting policy options to expand public health insurance coverage was significantly associated with a higher probability of early prenatal care initiation and lower probability of delayed initiation and receiving no prenatal care until delivery.

The estimates suggest that adopting State-only funds was associated with 87.1 percent points increase in initiating prenatal care in the first trimester (Odd ration [OR]: 1.871; 95 % CI: 1.622,2.159), 43.8 percent points decrease in delaying prenatal care (OR: 0.562; 95 % CI: 0.430,0.736), and 67.3 percent points decrease in not receiving prenatal care until delivery (OR: 0.327; 95 % CI: 0.204,0.522) for publicly insured immigrants compared to uninsured immigrants. After adopting Unborn Child option, the odds of initiating prenatal care in the second trimester increased by 34.4 % (OR: 1.344; 95 % CI: 0.983,1.130) and the odds of not receiving prenatal care until delivery decreased by 35.7 % (OR: 0.643; 95 % CI: 0.800,1.089) for publicly insured immigrants compared to uninsured immigrants.

However, the estimates suggest that adopting ICHIA option was not associated with early initiation of prenatal care among publicly insured immigrants. The sign of the association was suggestive of positive effects in both the first trimester and second trimester, but the point estimates were not statistically significant. On the contrary, adopting ICHIA option was associated with 28.8 percent point decrease in delay of prenatal care initiation (OR: 0.718; 95 % CI: 0.539,0.956) and 27.6 percent point decrease in not receiving prenatal care until delivery (OR: 0.643; 95 % CI: 0.800,1.089) for publicly insured immigrants compared to uninsured immigrants.

This study also found the association between mothers’ individual characteristics and prenatal care initiation. Older maternal age and education beyond high school were significantly associated with higher rates of early prenatal care initiation and lower rates of both delayed initiation and no prenatal care until delivery, though these effects were small. Non-Hispanic Asian mothers and firstborn children were associated with reduced delays and higher likelihood of prenatal care initiation before delivery. Whereas Non-Hispanic Black mothers and mothers identifying as AIAN or NHOPI were less likely to initiate care in the first trimester and more likely to delay or not receiving prenatal care until delivery.

## 4 Discussion

This study examined the effects of state policies providing public health insurance coverage for immigrant pregnant women on the initiation and adequacy of prenatal care, comparing outcomes between publicly insured and uninsured individuals. The findings indicated that all three state policy options were associated with reduced delays in initiating prenatal care among immigrant pregnant individuals. Previous research argued that extending Medicaid eligibility to immigrant families increased public insurance coverage and improved prenatal care (Chu et al., 2022; Choi et al., 2023). However, the results of this study did not confirm that the adoption of the ICHIA option significantly increase early initiation of prenatal care among publicly insured immigrants. This could be due to administrative challenges within the Medicaid program, as the ICHIA and FCEP channel funds through state Medicaid programs. High transaction costs and delays related to Medicaid enrollment can limit timely access to services (Moynihan et al., 2015; Fox et al., 2023). These administrative burdens might prevent immigrant pregnant women from scheduling their first prenatal visit in the first trimester due to delayed enrollment. Wherry at al. (2017) similarly found that eliminating the five-year waiting period and extending coverage to pregnancy from conception to the end of pregnancy did not significantly impact early prenatal care initiation among immigrant pregnant individuals.

This study found that the State-only funding option was positively associated with early prenatal care initiation. Unlike federally funded programs like Medicaid or CHIP, State-only funds operate independently from federal immigration oversight, minimizing the risk of immigration status complications (O'Connor, 2021; Wilson and Stimpson, 2020). This separation may make State-only funds more appealing to immigrants, who might feel more secure using these resources without potential immigration repercussions, especially given concerns around the “Public Charge Inadmissibility Rule” during the study period (Barofsky et al., 2020; Bernstein et al., 2020; Adams, 2018). Reduced immigration-related concerns may thus encourage earlier engagement with prenatal care. We also confirmed that State-only funds were associated with improved access to adequate prenatal care among publicly insured immigrants compared to uninsured immigrants. States with state-only funding tend to have stronger organizational connections among providers, clinics, and payors, potentially leading to improved service delivery for high-risk populations (Vesely et al., 2012). Such enhanced coordination can facilitate more appropriate and sustainable service utilization among vulnerable populations (Williams et al., 2019; Brewster et al., 2018).

This study contributes new evidence that policies expanding health insurance coverage for immigrant pregnant women have differential impacts on prenatal care initiation and utilization. State adoption of all three policies was associated with reduced delays in prenatal care, but the State-only funds option was linked to adequate prenatal care utilization. Immigrant families often face challenges with eligibility determination, complex application processes, and understanding covered benefits (Herd et al., 2013). Despite advances in modernized application methods, verifying income and immigration status prior to Medicaid enrollment remains difficult and time-sensitive (Fox et al., 2020; Camillo, 2021). Although electronic data matching could simplify eligibility verification, it requires data-sharing agreements (Brooks et al., 2015). Furthermore, renewing coverage is time-intensive due to automated system upgrades needed under ACA reforms (Camillo, 2021). These time-consuming administrative burdens may hinder timely access to prenatal care among immigrant families.

The study acknowledges limitations. First, insufficient tests for robustness of the main results in the research design may introduce a risk of unobserved biases (Gibson and Zimmerman, 2021). Second, the dataset lacks information on respondents’ financial status, limiting the analysis of eligibility criteria. Third, the dataset has no information on whether insurance coverage was in place prior to conception, affecting our ability to differentiate between pre- and post-conception coverage. Lastly, the three policy options are not mutually exclusive, allowing states to implement multiple policies simultaneously. This may complicate the process of isolating their individual effects on access to prenatal care. Future research could explore potential heterogeneity in policy effects, including differences across populations or geographies, by employing more flexible modeling approaches like interaction models or random coefficient frameworks that can accommodate such variation.

With immigration policy increasingly prominent on the national agenda in U.S., renewed attention to the impacts of health insurance coverage on prenatal care access is timely and essential. States attempted to maintain continuous Medicaid coverage during the pandemic, but the “unwinding” process beginning in 2023, which involves re-determined eligibility of existing beneficiaries, risks disenrolling many who may become uninsured (Dague and Ukert, 2024). Concerns over loss of health insurance are particularly pronounced given the administrative challenges on enrollment and renewal process of the program discussed earlier, which may have delay access to care.

Considering the significance of timely and adequate prenatal care initiation for maternal and infant health, federal and state agencies should address further disparities by offering continuous public health insurance to immigrants with streamlined enrollment and renewal processes, improving communication barrier, and incentivizing program participation.

## Funding statement

The authors did not receive support from any organization for the submitted work.

## Data statement

Following the data use agreement with the National Center for Health Statistics, raw data would remain confidential and will not be shared.

## Declaration of generative AI and AI-assisted technologies in the writing process

During the preparation of this work the author(s) used ChatGPT in order to improve readability. After using this tool/service, the author(s) reviewed and edited the content as needed and took full responsibility for the content of the publication.

## CRediT authorship contribution statement

**Gunah Kim:** Writing – original draft, Software, Methodology, Formal analysis, Data curation, Conceptualization. **S. Wilton Choi:** Writing – review & editing, Methodology, Formal analysis, Data curation. **Younhee Kim:** Writing – review & editing, Supervision.

## Declaration of competing interest

The authors declare that they have no known competing financial interests or personal relationships that could have appeared to influence the work reported in this paper.
